# Targeting Galectin-1 with Triptolide Induces Ferroptosis in Oral Squamous Cell Carcinoma

**DOI:** 10.3390/cancers18050782

**Published:** 2026-02-28

**Authors:** Wei-Tso Chia, Cheng-Yu Yang, Wei-Chin Chang, Chang-Huei Tsao, Chih-Kung Lin, Sien-Lin Ho, Chin-Shan Kuo, Chi-Tsung Wu, Ching-Hsien Tsai, Yu-Hsuan Li, Kuei-Yuan Chen, Gu-Jiun Lin, Chun-Shu Lin, Cheng-Chih Hsieh, Yuan-Wu Chen

**Affiliations:** 1 Department of Orthopedics, National Taiwan University Hospital Hsin-Chu Branch, Hsinchu 302, Taiwan; 4926602@yahoo.com.tw; 2 Department of Nursing, Yuan Pie University of Medical Technology, Hsinchu 302, Taiwan; 3 Department of Orthopaedic Surgery, Tri-Service General Hospital, Taipei 114, Taiwan; 4 School of Dentistry, National Defense Medical University, Taipei 114, Taiwan; hslcuyang@gmail.com (C.-Y.Y.); dentistapple@gmail.com (W.-C.C.); injection63@gmail.com (S.-L.H.); chinshan@mail.ndmctsgh.edu.tw (C.-S.K.); tina640114@yahoo.com.tw (C.-T.W.); sophia0410@gmail.com (C.-H.T.); e208017@gmail.com (Y.-H.L.); 0810ken@yahoo.com.tw (K.-Y.C.); 5 Department of Oral and Maxillofacial Surgery, Tri-Service General Hospital, Taipei 114, Taiwan; 6 Department and Graduate Institute of Microbiology and Immunology, National Defense Medical University, Taipei 114, Taiwan; changhuei@gmail.com; 7 Department of Medical Research, Tri-Service General Hospital, Taipei 114, Taiwan; 8 Division of Anatomic Pathology, Taipei Tzu Chi Hospital, New Taipei City 231, Taiwan; doc31795@yahoo.com.tw; 9 Department of Biology and Anatomy, National Defense Medical University, Taipei 114, Taiwan; lingujiun@mail.ndmctsgh.edu.tw; 10 Department of Radiation Oncology, Tri-Service General Hospital, National Defense Medical University, Taipei 114, Taiwan; chunshulin@gmail.com; 11 Department of Pharmacy, Kaohsiung Veterans General Hospital, Kaohsiung 813, Taiwan; 12 School of Pharmacy and Institute of Pharmacy, National Defense Medical University, Taipei 114, Taiwan; 13 Denway International Dental Hospital, Neihu, Taipei 114, Taiwan

**Keywords:** triptolide, Galectin-1, ferroptosis, oral squamous cell carcinoma, GPX4

## Abstract

**Background**: Oral squamous cell carcinoma (OSCC) remains clinically challenging, particularly in advanced disease, where treatment resistance limits therapeutic outcomes. Ferroptosis, an iron-dependent form of regulated cell death driven by lipid peroxidation, has emerged as a potential anticancer vulnerability. Galectin-1 (Gal-1/LGALS1), a β-galactoside–binding lectin frequently overexpressed in OSCC, is associated with tumor progression and unfavorable prognosis; however, its involvement in ferroptosis regulation remains incompletely understood. **Methods**: To investigate whether Triptolide (TPL) influences ferroptosis-associated responses through Gal-1 modulation, OSCC cell lines (SAS and HSC-3) were treated with TPL and analyzed for cell viability, lipid reactive oxygen species (ROS) accumulation, and glutathione peroxidase 4 (GPX4) expression. Publicly available The Cancer Genome Atlas (TCGA) datasets were examined to evaluate Gal-1 expression patterns and survival associations. An OSCC xenograft mouse model was further used to assess the antitumor effects of TPL and changes in ferroptosis-related markers in vivo. **Results**: TPL treatment reduced cell viability and increased lipid ROS accumulation in OSCC cells, accompanied by downregulation of GPX4 expression. Gal-1 expression was also decreased following TPL exposure in vitro and in xenograft tumors. Analysis of TCGA data revealed that elevated Gal-1 expression was significantly associated with poorer overall survival in OSCC patients. **Conclusions**: These findings indicate that TPL induces ferroptosis-associated responses in OSCC and suggest that this effect is partly mediated through modulation of Gal-1 expression. Gal-1 may represent a clinically relevant factor influencing ferroptosis susceptibility, and targeting this pathway warrants further investigation as a potential therapeutic strategy for OSCC.

## Simple Summary

Oral squamous cell carcinoma (OSCC) remains a challenging malignancy, particularly in advanced stages where current treatments are often ineffective. In this study, we investigated the therapeutic potential of Triptolide (TPL), a natural compound, in OSCC. Our results show that TPL induces ferroptosis-associated cell death by increasing lipid peroxidation and suppressing Galectin-1, a protein frequently overexpressed in OSCC and associated with poor clinical outcomes. Evidence from cell-based experiments, animal models, and patient tissue analyses supports a role for TPL in inhibiting tumor growth and modulating ferroptosis-related markers. Collectively, these findings suggest that targeting Galectin-1 may enhance ferroptosis susceptibility and represent a potential therapeutic strategy for OSCC.

## 1. Introduction

Oral squamous cell carcinoma (OSCC) represents the dominant subtype of head and neck cancers and remains a critical global health burden. Despite advances in clinical management, including surgery, radiotherapy, immunotherapy, and platinum-based chemotherapy, survival outcomes have shown minimal improvement over the past decades. Tumor recurrence, metastasis, and treatment resistance continue to hinder therapeutic success, leaving the overall 5-year survival rate stagnating around 50–60% [1,2]. The high incidence of locoregional recurrence, treatment resistance, and metastatic progression significantly contributes to this dismal prognosis [3]. In particular, the emergence of cisplatin resistance has become a central challenge in clinical oncology, urging the search for novel mechanisms and therapeutic strategies capable of circumventing drug resistance and enhancing tumor vulnerability.

Ferroptosis is a mechanistically distinct form of regulated cell death driven by iron accumulation and excessive lipid peroxidation. Unlike apoptosis or necroptosis, ferroptosis is defined by glutathione depletion, dysregulated antioxidant defenses, and inactivation of lipid peroxide–detoxifying enzymes such as GPX4. Increasing evidence suggests that ferroptosis plays a crucial role in cancer metabolism, therapeutic responses, and redox vulnerability [4]. Unlike apoptosis or necroptosis, ferroptosis is driven by oxidative stress and impaired lipid metabolism, typically through inhibition of glutathione peroxidase 4 (GPX4) and the Cystine/Glutamate transporter solute carrier family 7 member 11 (SLC7A11). The resultant buildup of reactive lipid species leads to irreversible membrane damage and cell death. Importantly, cancer cells under metabolic stress or with altered redox homeostasis often exhibit increased susceptibility to ferroptosis, making it an attractive strategy for targeting drug-resistant tumors [5,6].

Natural compounds with the capacity to induce ferroptosis have garnered considerable attention for their potential as adjunct or alternative therapies in cancer. Among them, Triptolide (TPL)—a diterpenoid triepoxide isolated from *Tripterygium wilfordii*—has demonstrated potent antitumor activity in various malignancies, including pancreatic, breast, lung, and hepatocellular cancers [7,8]. TPL has been shown to induce apoptosis, pyroptosis, and autophagy depending on context, but its role in modulating ferroptosis remains underexplored, especially in OSCC. In recent studies, TPL has been shown to suppress GPX4 expression and induce ferroptosis in cervical cancer and hepatocellular carcinoma [9,10], suggesting a conserved pathway that may extend to oral cancers.

Galectin-1 (Gal-1), a β-galactoside-binding lectin encoded by *LGALS1*, is frequently overexpressed in a wide range of tumors, including OSCC [11,12]. Gal-1 plays multifaceted roles in cancer progression, immune escape, angiogenesis, and drug resistance. Of note, emerging evidence links Gal-1 to redox modulation and ferroptosis regulation. In hepatocellular carcinoma, Gal-1 was reported to enhance resistance to sorafenib by suppressing ferroptosis through hepatocyte growth factor receptor (MET)/ AXL receptor tyrosine kinase (AXL) signaling and antioxidant control [13]. However, whether Gal-1 functions as a direct regulator of ferroptosis or simply modulates redox balance indirectly remains an open question. More importantly, its role in OSCC ferroptosis regulation has not been elucidated.

In our preliminary bioinformatics analyses using The Cancer Genome Atlas (TCGA), we observed that Gal-1 is highly expressed in OSCC tumors and its elevated expression correlates with significantly worse overall survival (log-rank *p* < 0.01), further supporting its clinical relevance as a prognostic marker. These findings underscore the importance of investigating Gal-1′s functional involvement in OSCC pathophysiology and treatment response.

Given this background, we hypothesize that Triptolide induces ferroptosis in OSCC cells via downregulation of Galectin-1. In this study, we employed a combination of molecular techniques—including cell viability assays, lipid ROS detection, Western blotting for GPX4, siRNA knockdown of *LGALS1*, and in vivo xenograft modeling—to determine whether Gal-1 serves as a functional mediator of TPL-induced ferroptosis. Our results reveal that Gal-1 suppression sensitizes OSCC cells to ferroptotic death, identifying it as a promising molecular target. This work expands the mechanistic understanding of TPL’s anticancer effects and supports the development of Gal-1–targeting ferroptosis strategies in OSCC.

## 2. Materials and Methods

### 2.1. TCGA Bioinformatics and Survival Analysis

Transcriptomic expression profiles quantified as transcripts per million (TPM) values and corresponding clinical metadata of the TCGA head and neck squamous cell carcinoma (TCGA-HNSC) cohort were obtained from the University of California, Santa Cruz, CA, USA (UCSC) Xena Browser (https://xenabrowser.net/, accessed on 17 July 2023) [14]. Data preprocessing and survival analyses were performed using R software (version 4.5.2; R Foundation for Statistical Computing, Vienna, Austria) within the RStudio environment (version 2025.09; Posit Software, Boston, MA, USA). Gene expression values were log2-transformed, and patients were stratified into high- and low-LGALS1 expression groups using maximally selected rank statistics implemented in the surv_cutpoint function of the survminer package, yielding an optimized cutoff value of TPM = 9.98.

Overall survival was analyzed using the Kaplan–Meier method, and differences between groups were assessed by the log-rank test. Survival time was truncated at 60 months (5 years) for survival visualization and comparison. UALCAN (University of Alabama at Birmingham CANcer data analysis Portal), an interactive web resource for cancer omics data analysis, was used to validate LGALS1 expression patterns and survival associations [15,16].

### 2.2. Clinical Specimens and Immunohistochemistry of Patient Tissues

Paired tumor and adjacent normal oral squamous cell carcinoma (OSCC) tissues (*n* = 73) were obtained from Tri-Service General Hospital (Taipei, Taiwan) with approval from the Institutional Review Board and written informed consent from all patients. Representative tumor and adjacent normal tissue cores were assembled into tissue microarrays (TMAs) using standard protocols. TMA sections (5 μm thick) were prepared from formalin-fixed, paraffin-embedded (FFPE) specimens. Sections were deparaffinized in xylene and rehydrated through a graded series of ethanol to distilled water. Antigen retrieval was performed by heating the sections in citrate buffer (10 mM, pH 6.0) using an electric heating device. After cooling to room temperature, endogenous peroxidase activity was quenched by incubation with 3% hydrogen peroxide for 10 min. The sections were then blocked to reduce nonspecific binding and incubated overnight at 4 °C with a primary antibody against Galectin-1 (1:500; GTX101566, GeneTex, Irvine, CA, USA). On the following day, sections were washed and incubated with an appropriate horseradish peroxidase–conjugated secondary antibody. Immunoreactivity was visualized using 3,3′-diaminobenzidine (DAB) as the chromogen, and nuclei were counterstained with hematoxylin. The stained sections were dehydrated, mounted, and examined under a light microscope. IHC evaluation was independently performed by a board-certified pathologist and a second observer trained in histopathologic assessment, both of whom were blinded to the clinical information. Staining intensity was scored on a scale of 0–3 (0, negative; 1, weak; 2, moderate; 3, strong), and the percentage of positively stained tumor cells was scored on a scale of 1–4 (1, 0–25%; 2, 26–50%; 3, 51–75%; 4, >75%). The final immunoreactivity score (I × P) was calculated by summing the intensity and percentage scores, yielding a total score ranging from 0 to 12. For statistical analyses, the average score of the two observers was used.

Clinical parameters were defined as follows: overall survival (OS) was defined as the time from diagnosis to death or last follow-up; local recurrence was defined as tumor reappearance at the primary site after treatment; and lymph node metastasis was defined as pathologically confirmed cervical lymph node involvement at the time of surgery. For clinicopathologic correlation and survival analyses, Galectin-1 expression was dichotomized into high and low expression groups using the mean I × P score (9.63) as the cutoff value.

### 2.3. Cell Lines and Culture Conditions

Human OSCC cell lines (SAS, SCC25, HSC-3) were obtained from authenticated biorepositories, including the Japanese Collection of Research Bioresources (JCRB, Osaka, Japan) and the Bioresource Collection and Research Center (BCRC, Hsinchu, Taiwan). Cells were grown in RPMI-1640 medium supplemented with 10% fetal bovine serum (FBS; biorion, Level Biotechnology, New Taipei City, Taiwan) and 1% antibiotic–antimycotic solution (containing 10,000 U/mL penicillin, 10 mg/mL streptomycin, and 25 µg/mL amphotericin B; Biological Industries, Beit HaEmek, Israel). Cultures were maintained at 37 °C in a humidified incubator with 5% CO_2_.

### 2.4. Drug Treatment and Reagents

Triptolide (TPL; Cayman Chemical, Ann Arbor, MI, USA) was dissolved in dissolved in dimethyl sulfoxide (DMSO) to prepare a stock solution and aliquots were stored at −20 °C to prevent degradation. Ferrostatin-1 (Fer-1; Cayman Chemical, Ann Arbor, MI, USA) was used as a ferroptosis rescue agent where indicated.

### 2.5. Cell Viability Assay

Cell viability was evaluated using a methylene blue-based colorimetric assay. Cells (5000/well) were treated with TPL for 24 or 48 h. Absorbance was measured at 570 nm, and viability was normalized to the vehicle control. Experiments were independently repeated three times (*n* = 3 biological replicates).

### 2.6. Lipid ROS Detection by Flow Cytometry

Lipid peroxidation was quantified using the oxidation-sensitive probe C11-BODIPY 581/591 (Thermo Fisher Scientific, Waltham, MA, USA). OSCC cells were exposed to TPL at the indicated concentrations for 48 h, followed by incubation with 2 µM C11-BODIPY in serum-free medium for 30 min at 37 °C in the dark. After staining, cells were washed with cold phosphate-buffered saline (PBS), resuspended in PBS containing 2% FBS, and analyzed immediately by flow cytometry (FACSCalibur, BD Biosciences, San Jose, CA, USA). A minimum of 20,000 events was collected for each sample. Lipid ROS induction was determined based on the characteristic fluorescence shift in oxidized C11-BODIPY from red (590 nm) to green (510 nm) emission. Flow cytometry data were processed using Kaluza Analysis Software (version1.2, Beckman Coulter, Brea, CA, USA). All experiments were performed in triplicate (*n* = 3 biological replicates).

### 2.7. Western Blotting and Densitometric Analysis

Total cellular protein was extracted using radioimmunoprecipitation assay (RIPA) lysis buffer supplemented with protease and phosphatase inhibitors (Thermo Fisher Scientific, Waltham, MA, USA). Equal protein amounts were separated by sodium dodecyl sulfate–polyacrylamide gel electrophoresis (SDS–PAGE) and transferred onto polyvinylidene difluoride (PVDF) membranes. After blocking, membranes were incubated overnight at 4 °C with primary antibodies against GPX4 (1:1000; ab125066, Abcam, Cambridge, UK), Galectin-1 (1:1000; GTX101566, GeneTex, Irvine, CA, USA), and glyceraldehyde-3-phosphate dehydrogenase (GAPDH) (1:10,000; ab181602, Abcam, Cambridge, UK) as the loading control. After washing, membranes were incubated with horseradish peroxidase (HRP)-conjugated secondary antibodies (1:5000; Abcam, Cambridge, UK) for 1 h at room temperature. Signals were detected using enhanced chemiluminescence (ECL; Thermo Fisher Scientific, Waltham, MA, USA). Band intensity was quantified using ImageJ (version 1.54r; National Institutes of Health, Bethesda, MD, USA). Target protein expression levels were normalized to GAPDH and expressed relative to the untreated control (defined as 1.0). Antibody dilutions were selected based on manufacturer recommendations and further optimized through preliminary titration experiments to ensure optimal signal-to-noise ratio. All Western blot analyses were performed using three independent biological replicates (*n* = 3).

### 2.8. Quantitative Real-Time PCR

Total RNA was extracted using TRIzol™ reagent (Invitrogen, Carlsbad, CA, USA), and RNA quality was assessed using a NanoDrop™ spectrophotometer (Thermo Fisher Scientific, Waltham, MA, USA). cDNA was synthesized from 1 μg RNA using the Maxima H Minus First Strand cDNA Synthesis Kit (Thermo Fisher Scientific, Waltham, MA, USA). qRT-PCR was performed using SYBR Green Master Mix (Thermo Fisher Scientific, Waltham, MA, USA) on a QuantStudio 5 system (Applied Biosystems, Foster City, CA, USA). Gene expression levels were calculated using the 2^−ΔΔCt^ method, with *GAPDH* as the reference gene. All assays were conducted in three independent biological replicates (*n* = 3). Primer sequences were designed using the Primer3-BLAST tool provided by the National Center for Biotechnology Information (NCBI, https://blast.ncbi.nlm.nih.gov/Blast.cgi; accessed on 17 July 2023) and synthesized by Genomics (Taipei, Taiwan). The following primers were used: LGALS1: Forward: 5′-AGCAGCGGGAGGCTGTCTTTC-3′, Reverse: 5′-ATCCATCTGGCAGCTTGACGGT-3′; GPX4: Forward: 5′-ACAAGAACGGCTGCGTGGTGAA-3′, Reverse: 5′-GCCACACACTTGTGGAGCTAGA-3′; SLC7A11: Forward: 5′-TCCTGCTTTGGCTCCATGAACG-3′, Reverse: 5′-AGAGGAGTGTGCTTGCGGACAT-3′; FTH1: Forward: 5′-TGAAGCTGCAGAACCAACGAGG-3′, Reverse: 5′-GCACACTCCATTGCATTCAGCC-3′; GAPDH: Forward: 5′-GTCTCCTCTGACTTCAACAGCG-3′, Reverse: 5′-ACCACCCTGTTGCTGTAGCCAA-3′.

### 2.9. Galectin-1 Knockdown and Overexpression

Galectin-1 knockdown was conducted using *LGALS1*-targeting siRNA (Dharmacon, Lafayette, CO, USA), with scrambled siRNA as the negative control. SAS and HSC-3 cells were transfected with 50 nM siRNA using DharmaFECT™ 1 transfection reagent (Dharmacon, Lafayette, CO, USA) in serum-free Opti-MEM (Gibco, Waltham, MA, USA), according to the manufacturer’s instructions. After 6 h, the medium was replaced with complete culture medium, and cells were harvested 48 h post-transfection for RNA and protein analysis. For overexpression, cells were transfected with a pCMV-Galectin-1 plasmid (OriGene, Rockville, MD, USA) using jetPRIME^®^ transfection reagent (Polyplus-transfection, Illkirch, France) following the manufacturer’s protocol. Expression efficiency for both knockdown and overexpression conditions was confirmed by qRT-PCR and Western blotting. All experiments were performed in three independent biological replicates (*n* = 3).

### 2.10. Ferroptosis Rescue and Mechanistic Experiments

To assess whether TPL-induced cytotoxicity was ferroptosis-dependent, cells were treated with Triptolide (TPL, 20 nM) alone or in combination with the ferroptosis inhibitor Ferrostatin-1 (Fer-1, 10 μM). Fer-1 was administered 1 h prior to TPL treatment and maintained throughout the incubation period. Following treatment for 48 h, rescue effects were evaluated using cell viability assays, lipid ROS measurement (C11-BODIPY staining), and Western blot analysis of ferroptosis-related proteins. A reduction in lipid peroxidation and recovery of GPX4 expression in the presence of Fer-1 were interpreted as evidence of ferroptosis involvement. All mechanistic rescue experiments were performed using three independent biological replicates (*n* = 3).

### 2.11. Xenograft Mouse Model

All animal experiments were conducted in accordance with institutional ethical regulations and approved by the Institutional Animal Care and Use Committee (IACUC approval #21-128). Six-week-old NOD-SCID mice (*n* = 5 per group) were subcutaneously injected with 2 × 10^6^ SAS cells suspended in PBS. Tumor formation was monitored until measurable masses reached approximately 80–100 mm^3^, after which mice were randomly assigned to either the control or treatment group. The treatment group received Triptolide (TPL, 0.15 mg/kg/day, intraperitoneally) for 14 consecutive days, while control mice received vehicle only. The dosing regimen was selected based on our previously reported OSCC xenograft model demonstrating therapeutic efficacy with acceptable systemic tolerance [17]. Body weight and general health status were assessed every 2–3 days to monitor potential toxicity. Tumor dimensions were measured using calipers, and tumor volume was calculated according to the standard formula: Tumor volume = (length × width^2^)/2. At study completion, mice were euthanized, and tumors were excised for subsequent histological and molecular analyses.

### 2.12. Immunohistochemistry of Xenograft Tissues

The immunohistochemistry procedure for xenograft tissues was performed separately from that used for human clinical specimens due to differences in tissue origin and antibody optimization conditions. Excised xenograft tumors were fixed in 10% neutral-buffered formalin for 24–48 h, embedded in paraffin, and sectioned at 5 μm thickness. Following deparaffinization and rehydration, antigen retrieval was performed by heating the sections in citrate buffer (10 mM, pH 6.0) using an electric heating device. Endogenous peroxidase activity was quenched using 3% hydrogen peroxide for 10 min. Sections were then blocked with 3% bovine serum albumin (BSA) and incubated overnight at 4 °C with primary antibodies against Galectin-1 (1:200; GeneTex) and GPX4 (1:200; Abcam). After washing, slides were incubated with an appropriate horseradish peroxidase–conjugated secondary antibody for 1 h at room temperature. Immunoreactivity was visualized using a DAB chromogenic substrate, and tissues were counterstained with hematoxylin, dehydrated, mounted, and imaged using a bright-field microscope (Olympus, Tokyo, Japan).

### 2.13. Statistical Analysis

All quantitative data are presented as mean ± standard deviation (SD) from at least three independent biological replicates. Statistical comparisons between two groups were performed using an unpaired two-tailed Student’s *t*-test, unless otherwise specified. For clinicopathological association analyses, chi-square (χ^2^) test or Fisher’s exact test was applied as appropriate. Kaplan–Meier survival analysis was performed using the log-rank test to compare survival differences between groups. A *p* value < 0.05 was considered statistically significant. All statistical analyses were conducted using GraphPad Prism (version 9; GraphPad Software, San Diego, CA, USA).

## 3. Results

### 3.1. Clinical Significance of Galectin-1 in OSCC

**Figure 1:**
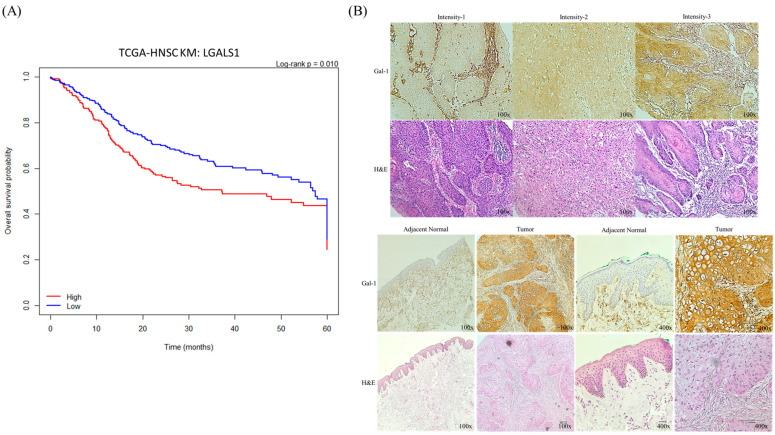
Galectin-1 is highly expressed in OSCC and is associated with adverse clinicopathologic features. (**A**) Kaplan–Meier overall survival analysis of the TCGA-HNSC cohort (*n* = 565) stratified by LGALS1 expression using an optimized cutpoint (TPM = 9.98), determined by maximally selected rank statistics. Survival time was truncated at 60 months (5 years). The high-expression group included 270 patients (47.8%), while the low-expression group included 295 patients (52.2%). Patients with high LGALS1 expression exhibited significantly shorter overall survival than those with low expression (log-rank *p* = 0.010). (**B**) Representative immunohistochemical (IHC) staining of Galectin-1 in human OSCC tissues and paired adjacent normal oral epithelium from a tissue microarray (TMA). Upper panels show representative tumor cores with variable staining intensities. Lower panels show two paired OSCC and adjacent normal cases demonstrating strong cytoplasmic Galectin-1 immunoreactivity in tumor tissues and weak or absent staining in normal epithelium (original magnification: ×100 and ×400). Associations between Galectin-1 IHC expression and clinicopathologic parameters are summarized in Table 1.

To determine the clinical relevance of Galectin-1 in oral squamous cell carcinoma (OSCC), we first analyzed transcriptomic expression profiles and clinical outcome data from the TCGA-HNSC cohort. Patients were stratified into high- or low-*LGALS1* expression groups using an optimized cutoff determined by maximally selected rank statistics (TPM = 9.98). Kaplan–Meier survival analysis revealed that patients with high *LGALS1* expression exhibited significantly poorer overall survival compared with those in the low-expression group (Figure 1A; log-rank *p* = 0.010). These results indicate that elevated LGALS1 expression is associated with unfavorable clinical outcomes in OSCC.

To validate these transcriptomic findings at the protein level, Galectin-1 expression was further examined by immunohistochemistry (IHC) in a tissue microarray (TMA) comprising paired OSCC tumor tissues and adjacent normal oral epithelium (*n* = 73). Representative staining images demonstrated stronger cytoplasmic Galectin-1 immunoreactivity in OSCC tissues compared with matched normal epithelium (Figure 1B). Semi-quantitative IHC scoring confirmed significantly higher Galectin-1 expression in tumor tissues (*p* < 0.05), consistent with the transcriptome-based analysis.

Clinicopathological correlation analysis based on IHC scores (Table 1) further demonstrated that high Galectin-1 expression was significantly associated with advanced tumor T category (T3–T4 vs. T1–T2, *p* = 0.021) and advanced AJCC stage (*p* = 0.018). In addition, patients with high Galectin-1 expression exhibited significantly poorer 5-year overall survival compared with those with low expression (*p* = 0.009). Collectively, these findings indicate that elevated Galectin-1 expression correlates with more aggressive tumor characteristics and adverse clinical outcomes in OSCC.

### 3.2. Triptolide Induces Ferroptosis Through Lipid ROS Accumulation and Downregulation of GPX4 in OSCC Cells

**Figure 2:**
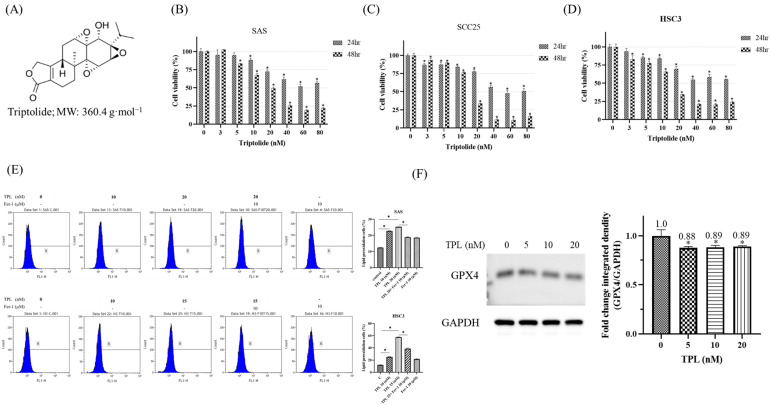
Triptolide (TPL) induces ferroptosis in OSCC cells by promoting lipid peroxidation and suppressing GPX4 expression. (**A**) Chemical structure of Triptolide (TPL). (**B**–**D**) Cell viability of OSCC cell lines SAS (**B**), SCC25 (**C**), and HSC-3 (**D**) treated with increasing concentrations of TPL (0–80 nM) for 24 and 48 h was measured using the methylene blue assay. (**E**) Lipid peroxidation was assessed using C11-BODIPY 581/591 staining followed by flow cytometry. SAS and HSC-3 cells were treated with TPL (20 nM, 48 h) alone or co-treated with Ferrostatin-1 (Fer-1, 10 μM). Fer-1 was added 1 h prior to TPL treatment. Right panel: quantitative analysis of lipid ROS-positive cells. Letters B denote the flow cytometric gating regions used to identify ROS-positive cell populations. (**F**) Western blot analysis showing GPX4 expression in SAS cells following TPL treatment (0–20 nM, 48 h). GAPDH served as the loading control. Densitometric quantification indicates a dose-dependent reduction in GPX4 expression. The GAPDH blot shown in Figure 2F is reused in Figure 3A because both panels were generated from the same experimental membrane under identical conditions. Data represent mean ± SD from three independent biological replicates (*n* = 3). * *p* < 0.05 compared with control. The original western blot figures can be found in Figure S1.

To determine whether Triptolide (chemical structure shown in Figure 2A) affects the survival of OSCC cells, SAS, SCC25, and HSC-3 cell lines were treated with increasing concentrations of TPL (0–80 nM) for 24 and 48 h, and cell viability was assessed using the methylene blue assay. TPL reduced cell viability in a dose- and time-dependent manner in all three cell lines (Figure 2B–D). These findings indicate that OSCC cells are sensitive to TPL treatment within nanomolar ranges. To assess whether ferroptosis contributes to TPL-induced cytotoxicity, lipid peroxidation was evaluated using the C11-BODIPY 581/591 fluorescent probe following 48 h of treatment. TPL treatment led to a marked increase in lipid ROS levels in both SAS and HSC-3 cells compared with vehicle-treated controls. Notably, co-treatment with the ferroptosis inhibitor Ferrostatin-1 (Fer-1, 10 μM) substantially attenuated lipid ROS accumulation (Figure 2E), suggesting involvement of lipid peroxidation processes. Given the established role of GPX4 as a key suppressor of ferroptosis, its expression was examined following TPL treatment. Western blot analysis demonstrated that GPX4 protein levels were reduced after 48 h of exposure to TPL in SAS cells (Figure 2F), and densitometric analysis confirmed statistically significant downregulation compared with untreated controls.

Taken together, these in vitro findings indicate that TPL triggers ferroptosis-associated molecular events in OSCC cells, characterized by increased lipid ROS and reduced GPX4 expression. While additional ferroptosis markers and broader validation in additional cell models will be needed to fully define the mechanistic relationship, these results support ferroptosis as a potential cell death pathway activated by TPL in OSCC.

### 3.3. Triptolide Downregulates Galectin-1 Expression in OSCC Cells

**Figure 3:**
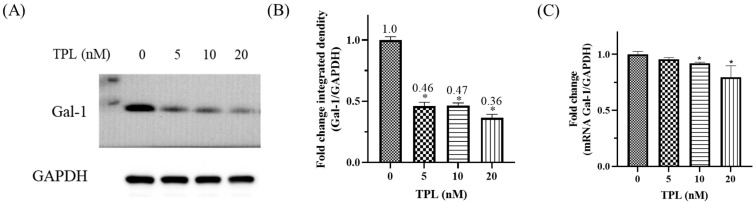
Triptolide suppresses Galectin-1 expression in OSCC cells. (**A**) Western blot analysis of Galectin-1 expression in SAS cells treated with increasing concentrations of Triptolide (TPL, 0–20 nM) for 48 h. GAPDH served as the loading control. (**B**) Densitometric quantification of Galectin-1 protein levels normalized to GAPDH and expressed relative to untreated control (set as 1.0). (**C**) Quantitative PCR (qPCR) analysis of LGALS1 mRNA expression in SAS cells following 48 h TPL treatment. Gene expression was normalized to GAPDH and calculated using the 2^−ΔΔCt^ method. The GAPDH loading control used in panel (**A**) is the same blot used in Figure 2F, as both analyses originated from the same membrane experiment. Data represent mean ± SD from three independent biological replicates (*n* = 3). * *p* < 0.05 compared with control. The original western blot figures can be found in Figure S2.

To assess whether Triptolide (TPL) regulates Galectin-1 expression in OSCC, SAS cells were treated with 20 nM TPL for 48 h and protein levels were examined by Western blotting. As shown in Figure 3A, TPL treatment resulted in a noticeable reduction in Galectin-1 protein expression compared with the vehicle control. Densitometric quantification confirmed that this decrease was statistically significant (Figure 3B, *p* < 0.05). To further investigate whether this regulatory effect occurred at the transcriptional level, LGALS1 mRNA expression was analyzed by quantitative real-time PCR. Consistent with the reduction observed at the protein level, TPL treatment significantly decreased LGALS1 transcript levels in SAS cells (Figure 3C).

Taken together, these findings indicate that Galectin-1 expression is responsive to TPL exposure at both protein and mRNA levels, suggesting transcriptional suppression as a potential mechanism. While the precise regulatory pathway remains to be determined, these results support the possibility that Galectin-1 downregulation may contribute to the cellular response to TPL in OSCC.

### 3.4. Galectin-1 Modulates Key Ferroptosis-Related Genes in OSCC Cells

**Figure 4:**
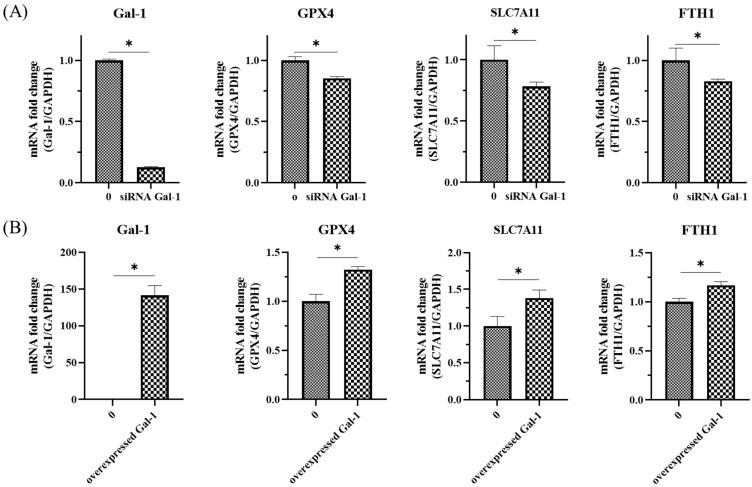
Galectin-1 regulates the expression of ferroptosis-associated genes in OSCC cells. (**A**) SAS cells were transfected with LGALS1-targeting siRNA (si-Gal-1) or scrambled control siRNA. After 48 h, mRNA expression levels of LGALS1, GPX4, SLC7A11, and FTH1 were measured using quantitative PCR (qPCR). (**B**) SAS cells were transfected with a pCMV-Galectin-1 overexpression plasmid. Following 48 h, the expression of LGALS1, GPX4, SLC7A11, and FTH1 was quantified by qPCR. Gene expression values were normalized to GAPDH and calculated using the 2^−ΔΔCt^ method. Data represent mean ± SD from three independent biological replicates (*n* = 3). * *p* < 0.05 compared with control.

To investigate the potential role of Galectin-1 in regulating ferroptosis-associated pathways, gain- and loss-of-function studies were conducted in SAS cells. In the knockdown model, siRNA targeting Galectin-1 (si-Gal-1) markedly reduced LGALS1 mRNA expression compared with the negative control (Figure 4A). Notably, the reduction in Galectin-1 expression was accompanied by significantly decreased expression levels of ferroptosis-associated genes, including GPX4, SLC7A11, and FTH1 (*p* < 0.05), all of which are recognized contributors to cellular antioxidant defense and ferroptosis resistance. Conversely, Galectin-1 overexpression via a pCMV-Gal-1 plasmid resulted in elevated LGALS1 transcript levels and correspondingly increased expression of GPX4, SLC7A11, and FTH1 (Figure 4B). The reciprocal expression patterns observed in knockdown and overexpression conditions support a functional association between Galectin-1 expression and the transcription of key ferroptosis-related genes.

Collectively, these findings suggest that Galectin-1 may contribute to ferroptosis resistance mechanisms in OSCC by modulating the expression of redox-regulatory genes. Additional mechanistic studies will be required to determine whether this occurs through direct transcriptional control or via upstream regulatory signaling pathways.

### 3.5. Effects of Galectin-1 Knockdown, Restoration, and Ferrostatin-1 Co-Treatment on TPL-Induced Ferroptosis

**Figure 5:**
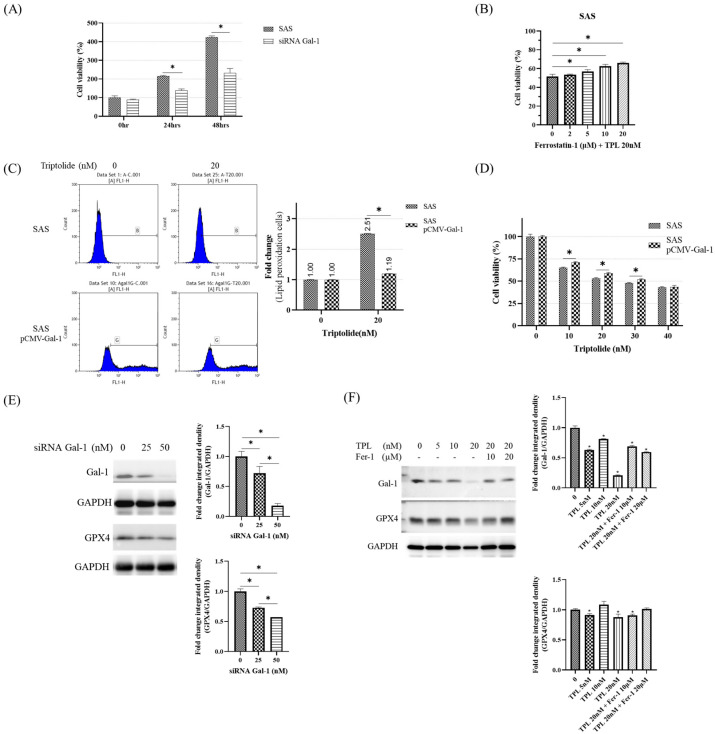
Functional involvement of Galectin-1 in TPL-induced ferroptosis in OSCC cells. (**A**) SAS cells were transfected with Galectin-1–targeting siRNA (si-Gal-1) or scrambled control for 48 h, and cell viability was assessed using the methylene blue assay. (**B**) SAS cells were treated with Triptolide (TPL, 20 nM) for 48 h in the presence or absence of Ferrostatin-1 (Fer-1, 10 μM), and cell viability was measured. (**C**) Lipid reactive oxygen species (ROS) levels were determined by C11-BODIPY 581/591 staining followed by flow cytometry in TPL-treated cells (20 nM, 48 h) with or without ectopic Galectin-1 expression achieved by transfection with pCMV-Gal-1. Left panel: representative histograms; right panel: quantitative analysis of lipid ROS–positive cells. Letters B and G denote the flow cytometric gating regions used to identify ROS-positive cell populations. (**D**) Cell viability of TPL-treated cells (20 nM, 48 h) transfected with pCMV-Gal-1 or control vector. (**E**) Western blot analysis of GPX4 expression following Galectin-1 knockdown. (**F**) Western blot analysis of Galectin-1 and GPX4 expression in cells treated with TPL (20 nM, 48 h) alone or in combination with Fer-1 (10 μM). Densitometric analysis was performed using ImageJ software, and protein expression levels were normalized to GAPDH. Data are presented as mean ± SD from three independent biological replicates (*n* = 3). * *p* < 0.05 compared with the respective control groups. The original western blot figures can be found in Figures S3 and S4.

To further investigate the functional contribution of Galectin-1 to ferroptosis susceptibility in OSCC cells, we examined the effects of genetic depletion, ectopic restoration, and pharmacological inhibition of lipid peroxidation on Triptolide (TPL)-induced cytotoxicity. Silencing Galectin-1 using siRNA significantly reduced SAS cell viability compared with scrambled controls (Figure 5A, *p* < 0.05), indicating that Galectin-1 contributes to the maintenance of OSCC cell survival. Consistent with earlier findings, TPL treatment markedly decreased cell viability, whereas co-treatment with the ferroptosis inhibitor Ferrostatin-1 (Fer-1) partially restored viability (Figure 5B), supporting the involvement of ferroptosis in TPL-induced cytotoxicity.

To directly determine whether Galectin-1 downregulation is functionally involved in TPL-induced ferroptotic responses, lipid peroxidation was evaluated using C11-BODIPY 581/591 staining followed by flow cytometry. TPL treatment induced a pronounced increase in lipid ROS levels, which was significantly attenuated by ectopic Galectin-1 expression achieved by transfection with pCMV-Gal-1 (Figure 5C). Consistently, restoration of Galectin-1 expression partially rescued cell viability in TPL-treated cells (Figure 5D), indicating that Galectin-1 downregulation contributes functionally to TPL-induced ferroptotic cell death. Similar attenuation of lipid ROS by Gal-1 restoration was also observed in HSC-3 cells (Supplementary Figure S5).

In support of these functional observations, Western blot analysis revealed that Galectin-1 knockdown markedly reduced GPX4 protein expression (Figure 5E). Moreover, TPL exposure decreased the expression of both Galectin-1 and GPX4, whereas Fer-1 co-treatment partially restored their levels (Figure 5F). This restoration is interpreted as a secondary consequence of improved cell survival under ferroptosis inhibition rather than evidence of a direct regulatory feedback mechanism.

Collectively, these results demonstrate that Galectin-1 modulates ferroptosis susceptibility in OSCC cells and that its suppression—either genetically or through TPL exposure—sensitizes cells to ferroptotic stress. The partial rescue observed upon Galectin-1 restoration further suggests that additional Galectin-1–independent mechanisms may also contribute to TPL-induced ferroptotic responses.

### 3.6. Triptolide Treatment Inhibits OSCC Tumor Growth and Modulates Ferroptosis-Associated Markers In Vivo

**Figure 6:**
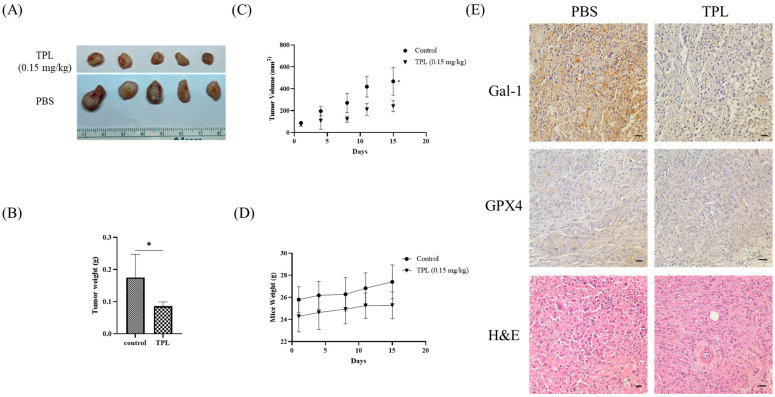
Triptolide suppresses tumor growth and modulates ferroptosis-related markers in OSCC xenograft models. (**A**) Representative images of SAS xenograft tumors harvested after treatment. Mice received either vehicle (PBS) or Triptolide (TPL, 0.15 mg/kg/day, intraperitoneally) for 14 days. (**B**) Final tumor weights at the end of treatment. (**C**) Tumor volumes measured every other day and calculated using the formula: Tumor volume = (length × width^2^)/2. (**D**) Body weight monitoring throughout the treatment period to assess systemic toxicity. (**E**) Representative immunohistochemical (IHC) staining of xenograft tumors for Galectin-1 and GPX4. Hematoxylin and eosin (H&E) staining shows tumor morphology. Magnification: 400×. Tumor growth experiments were performed with five mice per group (*n* = 5). Data are presented as mean ± SD. * *p* < 0.05 compared with vehicle control.

To evaluate the therapeutic effects of Triptolide (TPL) in vivo, SAS xenograft tumors were established in NOD-SCID mice and treated with TPL (0.15 mg/kg/day, intraperitoneally) or vehicle control for 14 days following tumor establishment. As shown in Figure 6A, tumors from TPL-treated mice appeared smaller compared with those from the control group. Quantitative measurements confirmed a significant reduction in tumor weight (Figure 6B, *p* < 0.01) and slower tumor volume progression throughout the treatment course (Figure 6C, *p* < 0.05). No significant differences in body weight were observed between treatment groups (Figure 6D), indicating that TPL was well tolerated under the tested dosage and schedule. To determine whether molecular alterations observed in vitro were reflected in vivo, immunohistochemistry (IHC) analysis was performed on xenograft tumor tissues. TPL-treated tumors displayed visibly reduced Galectin-1 and GPX4 expression compared with vehicle controls (Figure 6E). The downregulation of these markers aligns with in vitro findings and suggests that ferroptosis-related pathways may be involved in the antitumor effects of TPL.

Together, these in vivo data indicate that TPL treatment suppresses OSCC tumor growth and is associated with reduced expression of Galectin-1 and GPX4. While these findings are consistent with ferroptosis-related molecular patterns observed in vitro, additional validation across multiple OSCC models and expanded ferroptosis marker panels will be required to further define the mechanistic contribution of ferroptosis to TPL’s antitumor activity.

## 4. Discussion

Ferroptosis has gained increasing attention as a therapeutic vulnerability in several malignancies, including oral squamous cell carcinoma (OSCC), where treatment resistance and poor survival rates remain major clinical challenges [5,18]. In this study, we provide evidence that Triptolide (TPL) induces ferroptosis-associated responses in OSCC cells, as indicated by reduced cell viability, elevated lipid ROS levels, and suppression of the ferroptosis-defense enzyme GPX4. These observations are consistent with established models in which GPX4 depletion promotes lipid peroxidation-dependent cell death [19].

A key finding of this study is that TPL significantly downregulates Galectin-1 (Gal-1) expression at both mRNA and protein levels. Gal-1, a β-galactoside-binding lectin, has been implicated in tumor immune evasion, angiogenesis, metastasis, and therapeutic resistance across multiple cancer types [20,21,22,23,24,25]. Consistent with previous reports [26], and our TCGA analyses, OSCC tissues with elevated Gal-1 expression exhibited poorer survival outcomes and were associated with more advanced tumor stages. These clinical correlations support the relevance of Gal-1 as a tumor-associated biomarker in OSCC.

Mechanistically, our gain- and loss-of-function assays demonstrated that Gal-1 modulates the expression of multiple ferroptosis-associated genes, including GPX4, SLC7A11, and FTH1. Silencing Gal-1 reduced the expression of these antioxidant defense components, whereas Gal-1 overexpression increased their levels. Importantly, restoration of Gal-1 expression partially attenuated TPL-induced lipid ROS accumulation and cell death, providing functional evidence that Gal-1 downregulation contributes to ferroptosis susceptibility. These findings suggest that Gal-1 plays a modulatory role in maintaining redox homeostasis and ferroptosis resistance in OSCC cells.

The clinical association between high Gal-1 expression and advanced tumor stage observed in our cohort (Table 1) suggests a potential functional role for Gal-1 in supporting tumor aggressiveness. OSCC progression is characterized by elevated oxidative stress, hypoxia, and metabolic imbalance within the tumor microenvironment. Under such conditions, cancer cells rely heavily on antioxidant defense mechanisms to preserve membrane integrity and prevent ferroptotic cell death. Given our findings that Gal-1 supports the expression of ferroptosis-protective genes, including GPX4, SLC7A11, and FTH1, it is plausible that elevated Gal-1 expression confers a selective survival advantage by limiting lipid peroxidation and ferroptosis. This ferroptosis-escape mechanism may contribute to tumor progression and help explain why tumors with high Gal-1 expression are more likely to develop advanced (T3–T4) disease.

Although the upstream regulatory mechanisms remain incompletely defined, prior studies suggest potential links between Gal-1 and NRF2-mediated antioxidant signaling [20,27]. NRF2 regulates several ferroptosis-protective genes, including GPX4, SLC7A11, and iron metabolism regulators [28,29]. Accordingly, a possible Gal-1–NRF2–GPX4/SLC7A11 regulatory axis may contribute to ferroptosis resistance in OSCC, although this hypothesis requires further experimental validation.

Notably, TPL has been reported to inhibit NRF2 signaling in other tumor models by reducing nuclear NRF2 levels and suppressing NRF2-dependent antioxidant genes [9,30,31]. In this context, our findings raise a plausible model in which TPL reduces ferroptosis resistance through simultaneous suppression of Gal-1 expression and NRF2-controlled antioxidant pathways. While this model is biologically consistent with our observations, further studies—including direct NRF2 modulation and promoter binding assays—are required to establish mechanistic causality.

Our functional data further demonstrated that Ferrostatin-1 partially rescued TPL-induced cytotoxicity and restored GPX4 and Gal-1 protein levels. Consistent with reviewer feedback, this restoration is interpreted as a secondary consequence of improved cellular survival under ferroptosis inhibition rather than evidence of a direct regulatory feedback loop. Thus, although our results support a functional association between Gal-1 and ferroptosis regulation, definitive causal relationships remain to be fully elucidated.

Our in vivo findings further support the relevance of this pathway. TPL significantly reduced tumor burden in OSCC xenograft models without detectable systemic toxicity, accompanied by decreased Gal-1 and GPX4 expression in tumor tissues. These results reinforce the translational relevance of Gal-1–associated ferroptosis regulation in OSCC.

These rescue effects were also validated in HSC-3 cells, supporting the robustness and reproducibility of the observed phenotype. Several limitations of this study should be acknowledged. First, ferroptosis was inferred primarily based on lipid ROS accumulation and GPX4 downregulation; additional biochemical and genetic approaches—such as iron chelation, lipid peroxidation assays, or GPX4 rescue—would further strengthen causal interpretation. Second, although key findings were validated in HSC-3 cells, most mechanistic experiments were performed in SAS cells; validation in additional OSCC models or patient-derived systems would improve generalizability. Finally, the relationship between Gal-1 and NRF2-regulated antioxidant pathways remains hypothetical and warrants targeted investigation.

Together, these findings suggest that TPL induces ferroptosis-associated vulnerability in OSCC and that Gal-1 functions as a molecular modulator influencing ferroptosis resistance. The proposed interaction among Gal-1, NRF2 signaling, and GPX4 regulation may represent a potential therapeutic axis for biomarker-guided ferroptosis-based strategies in OSCC.

## 5. Conclusions

In summary, this study provides evidence that Triptolide (TPL) induces ferroptosis-associated responses in oral squamous cell carcinoma (OSCC), characterized by lipid ROS accumulation, reduced cell viability, and suppression of the ferroptosis-related enzyme GPX4. We further show that TPL downregulates Galectin-1 at both transcriptional and protein levels, and that modulation of Galectin-1 influences the expression of key ferroptosis-associated genes, including GPX4, SLC7A11, and FTH1. These findings suggest that Galectin-1 may serve as a modulatory factor contributing to ferroptosis resistance mechanisms in OSCC.

Consistent with these mechanistic observations, TPL significantly suppressed tumor growth in OSCC xenograft models and reduced Galectin-1 and GPX4 expression in tumor tissues. Together with clinical dataset analyses demonstrating that elevated Galectin-1 expression correlates with poor survival and advanced disease stage, these results support the potential relevance of Galectin-1 as a prognostic biomarker and therapeutic target.

While these findings broaden the understanding of ferroptosis vulnerability in OSCC, additional studies—including pathway dissection, broader ferroptosis marker validation, and genetic rescue experiments—will be required to define the mechanistic hierarchy between Galectin-1, ferroptosis-related genes, and TPL responsiveness. Nonetheless, this work provides a rationale for further exploration of Galectin-1–directed ferroptosis sensitization strategies and supports continued investigation of TPL and related agents as candidates for ferroptosis-based therapeutic development in OSCC.

## Figures and Tables

**Table 1:** Clinicopathological characteristics of OSCC patients according to Galectin-1 expression (*n =* 73).

Galectin-1 Expression Was Dichotomized Using the Mean I × P Score (9.63).				
Clinicopathological Parameters	Total (*n* = 73)	Gal-1 Low (*n* = 36)	Gal-1 High (*n* = 37)	*p* Value
**Age (years)**	50.8 ± 9.6	50.1 ± 9.8	51.4 ± 9.5	0.58
**Gender**				0.74
Male	61 (83.6%)	30 (83.3%)	31 (83.8%)	
Female	12 (16.4%)	6 (16.7%)	6 (16.2%)	
**Tumor site**				0.81
Buccal	25 (34.2%)	13	12	
Tongue	26 (35.6%)	12	14	
Gingiva	12 (16.4%)	6	6	
Others	10 (13.8%)	5	5	
**Tumor size (T)**				**0.021**
T1–T2	30 (41.1%)	19	11	
T3–T4	43 (58.9%)	17	26	
**Lymph node metastasis (pathological)**				0.11
LN(−)	33 (45.2%)	19	14	
LN(+)	40 (54.8%)	17	23	
**AJCC stage**				**0.018**
I–II	24 (32.9%)	16	8	
III–IV	49 (67.1%)	20	29	
**Pathological grade**				0.62
Grade 1–2	48 (65.8%)	25	23	
Grade 3	25 (34.2%)	11	14	
**5-year overall survival**				**0.009**
Alive at 5 years	46 (63.0%)	28	18	
Dead within 5 years	27 (37.0%)	8	19	

Galectin-1 expression was assessed by semi-quantitative immunohistochemical (IHC) scoring using an intensity × percentage (I × P) system. Patients were dichotomized into Galectin-1–low and Galectin-1–high groups using the mean I × P score (cutoff = 9.63). The associations between Galectin-1 expression status and clinicopathologic parameters were statistically analyzed as summarized in this table. Lymph node (LN) status was determined based on pathological assessment at surgery. *p* values were calculated using the chi-square (χ^2^) test or Fisher’s exact test, as appropriate. Bold values indicate statistically significant differences (*p* < 0.05).

## Data Availability

The data that support the findings of this study are available from the corresponding authors upon reasonable request.

## Supplementary Materials

The following supporting information can be downloaded at: https://www.mdpi.com/article/10.3390/cancers18050782/s1, Figure S1: Panel A represents the original western blot in Figure 2F; Figure S2: Panel B represents the original western blot in Figure 3A; Figure S3: Panel C represents the original western blot in Figure 5E; Figure S4: Panel C represents the original western blot in Figure 5F; Figure S5: Validation of Galectin-1–mediated regulation of lipid ROS in HSC-3 cells. Lipid reactive oxygen species (ROS) levels were assessed by C11-BODIPY 581/591 staining followed by flow cytometry in HSC3 cells treated with TPL (15 nM, 48 h) with or without Galectin-1 overexpression achieved by transfection with a Galectin-1 expression plasmid (pCMV-Gal-1). Left panel: representative flow cytometry histograms; right panel: quantitative analysis of lipid ROS–positive cells. * *p* < 0.05 compared with respective controls.
