# Supplementary material for: Targeting Galectin-1 with Triptolide Induces Ferroptosis in Oral Squamous Cell Carcinoma
Source: Cancers (Basel). 2026 Feb 28;18(5):782. doi: 10.3390/cancers18050782 (PMC12984301; doi:10.3390/cancers18050782)
Supplement: Supplementary file 1 [file cancers-18-00782-s001.zip › cancers-4148259-supplementary.pdf]

Panel A represents the original western blot in Fig 2F.

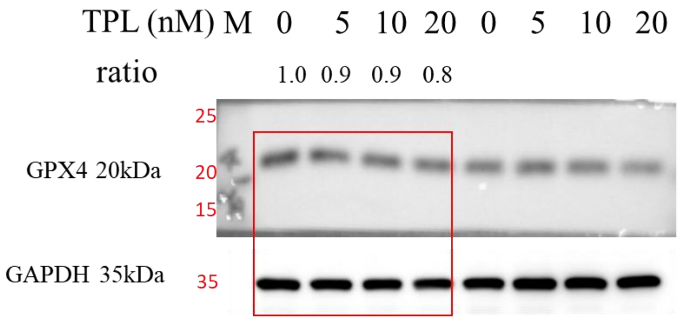

Supplementary Figure S1. Panel A represents the original western blot in Figure 2F.

Panel B represents the original western blot in Fig 3A.

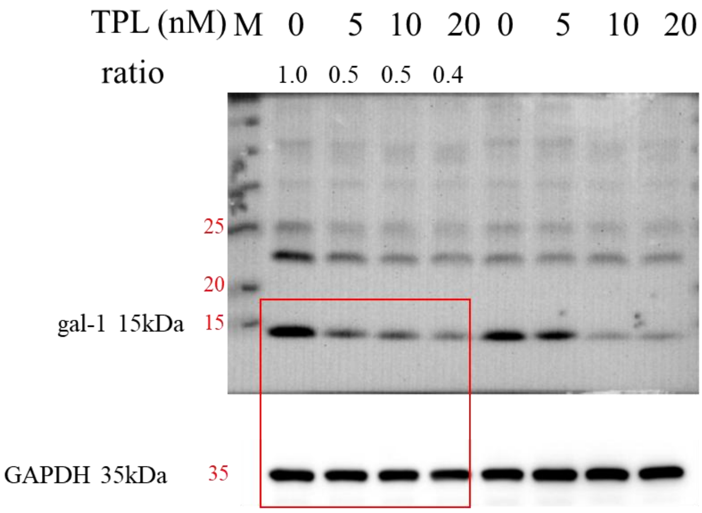

Supplementary Figure S2. Panel B represents the original western blot in Figure 3A.

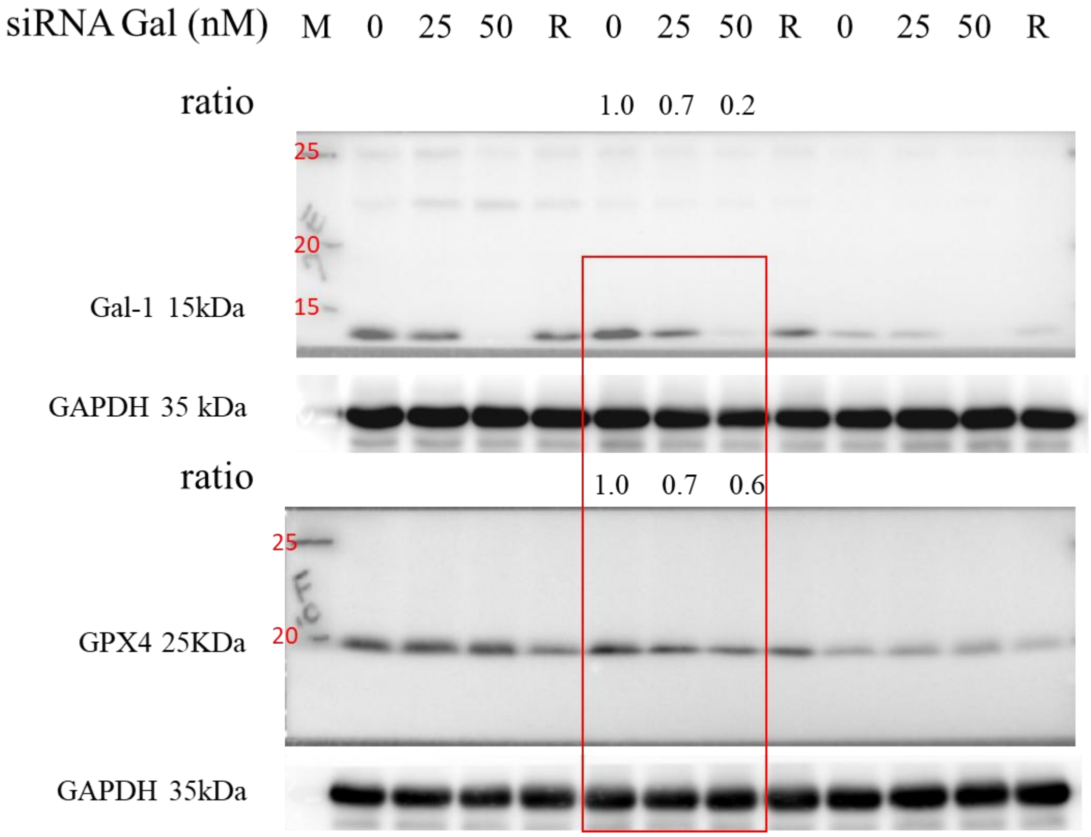

Supplementary Figure S3. Panel C represents the original western blot in Figure 5E.

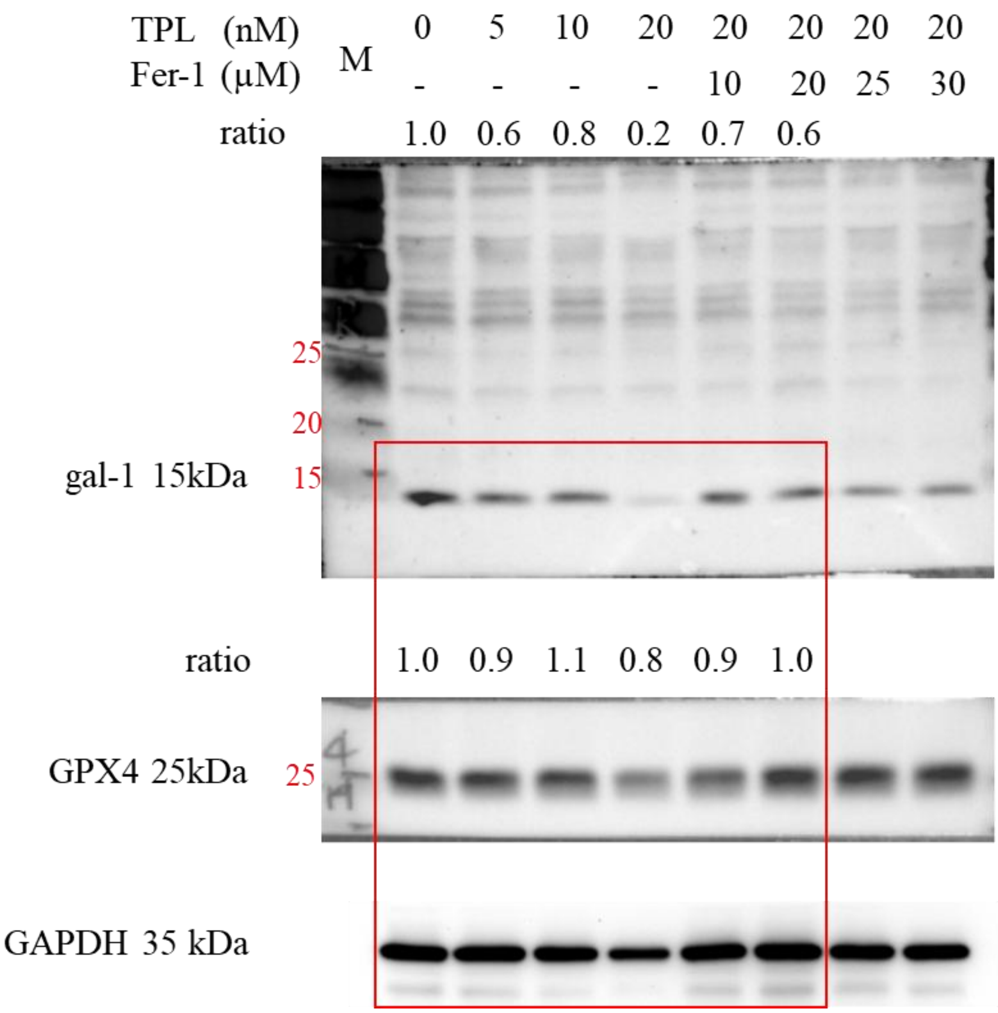

Supplementary Figure S4. Panel C represents the original western blot in Figure 5F.

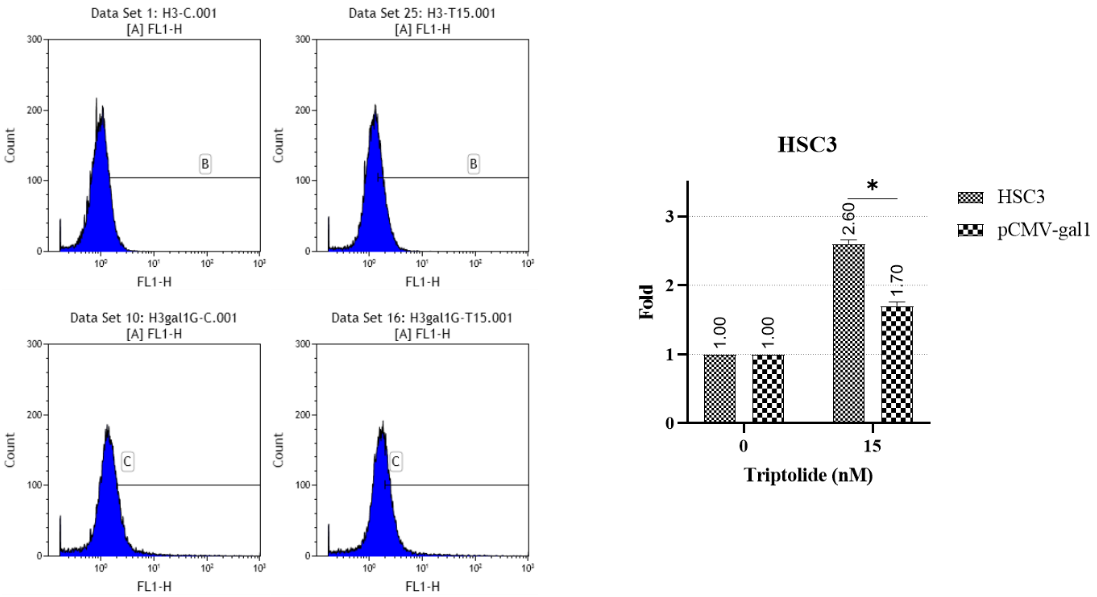

Supplementary Figure S5. Validation of Galectin-1–mediated regulation of lipid ROS in HSC-3 cells. Lipid reactive oxygen species (ROS) levels were assessed by C11-BODIPY 581/591 staining followed by flow cytometry in HSC3 cells treated with TPL (15 nM, 48 h) with or without Galectin-1 overexpression achieved by transfection with a Galectin-1 expression plasmid (pCMV-Gal-1). Left panel: representative flow cytometry histograms; right panel: quantitative analysis of lipid ROS–positive cells. *$p < 0.05$ compared with respective controls.
